# Correcting mortality estimates among children and youth on antiretroviral therapy in southern Africa: A comparative analysis between a multi-country tracing study and linkage to a health information exchange

**DOI:** 10.1111/tmi.14030

**Published:** 2024-07-04

**Authors:** Patience Nyakato, Michael Schomaker, Andrew Boulle, Jonathan Euvrard, Robin Wood, Brian Eley, Hans Prozesky, Benedikt Christ, Nanina Anderegg, Irene Ayakaka, Idiovino Rafael, Cordelia Kunzekwenyika, Carolyn B. Moore, Monique van Lettow, Cleophas Chimbetete, Safari Mbewe, Marie Ballif, Matthias Egger, Constantin T. Yiannoutsos, Morna Cornell, Mary-Ann Davies

**Affiliations:** 1 Centre for Infectious Disease Epidemiology and Research, School of Public Health, Faculty of Health Sciences, University of Cape Town, Cape Town, South Africa; 2 Wellcome Centre for Infectious Diseases Research in Africa, Institute of Infectious Disease and Molecular Medicine, Faculty of Health Sciences, University of Cape Town, Cape Town, South Africa; 3 Department of Statistics, Ludwig-Maximilians-Universität München, Munich, Germany; 4 Khayelitsha ART Programme, Cape Town, South Africa; 5 Western Cape Government: Health and Wellness, Cape Town, South Africa; 6 Gugulethu HIV Programme and Desmond Tutu HIV Centre, University of Cape Town, Cape Town, South Africa; 7 Red Cross War Memorial Children’s Hospital and Department of Paediatrics and Child Health, University of Cape Town, Cape Town, South Africa; 8 Division of Infectious Diseases, Department of Medicine, University of Stellenbosch and Tygerberg Academic Hospital, Cape Town, South Africa; 9 Institute of Social and Preventive Medicine, University of Bern, Bern, Switzerland; 10 SolidarMed, Maseru, Lesotho; 11 SolidarMed, Pemba, Mozambique; 12 SolidarMed, Masvingo, Zimbabwe; 13 Centre for Infectious Diseases Research in Zambia, Lusaka, Zambia; 14 Dignitas International, Zomba, Malawi; 15 Madiro, Toronto, Canada; 16 Dalla Lana School of Public Health, University of Toronto, Toronto, Canada; 17 Newlands Clinic, Harare, Zimbabwe; 18 Lighthouse Trust Clinic, Lilongwe, Malawi; 19 Department of Infectious Diseases, Inselspital, Bern University Hospital, University of Bern, Bern, Switzerland; 20 R.M Fairbanks, School of Public Health, Department of Biostatistics, Indiana University, Indianapolis, Indiana, USA

**Keywords:** children and youth, linkage, loss to follow-up, mortality, self-transfer, tracing

## Abstract

**Objectives:**

The objective of this study is to assess the outcomes of children, adolescents and young adults with HIV reported as lost to follow-up, correct mortality estimates for children, adolescents and young adults with HIV for unascertained outcomes in those loss to follow-up (LTFU) based on tracing and linkage data separately using data from the International epidemiology Databases to Evaluate AIDS in Southern Africa.

**Methods:**

We included data from two different populations of children, adolescents and young adults with HIV; (1) clinical data from children, adolescents and young adults with HIV aged ≤24 years from Lesotho, Malawi, Mozambique, Zambia and Zimbabwe; (2) clinical data from children, adolescents and young adults with HIV aged ≤14 years from the Western Cape (WC) in South Africa. Outcomes of patients lost to follow-up were available from (1) a tracing study and (2) linkage to a health information exchange. For both populations, we compared six methods for correcting mortality estimates for all children, adolescents and young adults with HIV.

**Results:**

We found substantial variations of mortality estimates among children, adolescents and young adults with HIV reported as lost to follow-up versus those retained in care. Ascertained mortality was higher among lost and traceable children, adolescents and young adults with HIV and lower among lost and linkable than those retained in care (mortality: 13.4% [traced] vs. 12.6% [retained-other Southern Africa countries]; 3.4% [linked] vs. 9.4% [retained-WC]). A high proportion of lost to follow-up children, adolescents and young adults with HIV had self-transferred (21.0% and 47.0%) in the traced and linked samples, respectively. The uncorrected method of non-informative censoring yielded the lowest mortality estimates among all methods for both tracing (6.0%) and linkage (4.0%) approaches at 2 years from ART start. Among corrected methods using ascertained data, multiple imputation, incorporating ascertained data (MI (asc.)) and inverse probability weighting with logistic weights were most robust for the tracing approach. In contrast, for the linkage approach, MI(asc.) was the most robust.

**Conclusions:**

Our findings emphasise that lost to follow-up is non-ignorable and both tracing and linkage improved outcome ascertainment: tracing identified substantial mortality in those reported as lost to follow-up, whereas linkage did not identify out-of-facility deaths, but showed that a large proportion of those reported as lost to follow-up were self-transfers.

## INTRODUCTION

Accurate estimation of mortality among children, adolescents and young adults with HIV (CAYHIV) is essential for HIV program evaluation. However, reported loss to follow-up (LTFU) remains a challenge as it may obscure several true outcomes. Mortality may differ between lost and retained patients, with many patients reported as LTFU having died [1]. Furthermore, some patients reported as lost at one facility may have self-transferred to a different facility and still be receiving care [2–5]. Not accounting for outcomes in those reported as LTFU may result in underestimation of mortality and retention [6–8], impacting policy decisions.

Despite complexities surrounding reported LTFU, most routine settings assess mortality and retention from facility-level information in paper-based and/or electronic patient databases, which may be incomplete/ inaccurate, especially without tracing of those reported as LTFU [9]. Site-reported mortality among adults with HIV may be underestimated by up to 50% when not accounting for mortality in those LTFU [10]. Limited data are available on outcomes among lost CAYHIV, who may have different reasons for appearing LTFU compared with adults due to dependence on caregivers and mobility for education or employment. A systematic review of retention of children with HIV in the first 12 months on ART reported that only 12/35 studies described efforts to trace LTFU children, and none documented outcomes of traced children [11].

To generate unbiased mortality estimates, we need data on outcomes in a representative sample of those LTFU. This can be obtained through tracing or linkage to other health information exchanges. These additional data need to be analysed alongside the outcomes of retained CAYHIV. Although there have been numerous analyses in adult populations [1, 2, 12], there is a dearth of research on CAYHIV. We assessed outcomes of CAYHIV reported as LTFU using two separate additional data samples obtained using (i) tracing of all CAYHIV (Aged <25 years) in five Southern African countries and (ii) linkage of all CAYHIV (Aged <15 years) in the Western Cape (WC), South Africa. We then corrected mortality estimates in the two populations where the samples were obtained separately and assessed predictors of mortality with six methods using data from the International epidemiology Database to Evaluate AIDS in Southern Africa (IeDEA-SA).

## METHODS

This study comprised two components (tracing- and linkage-informed studies) utilising data from six Southern African countries with ART cohorts that contribute to IeDEA-SA. IeDEA-SA is one of seven regions in the global IeDEA consortium that collects and harmonises HIV/AIDS data [13, 14]. IeDEA collects observational data from clinical centres and research groups in 44 countries, representing over 2.2 million people living with and at risk of HIV [13–15]. These data are organised into 7 geographic regions and coordinated by centres for Asia-Pacific, the Caribbean, Central and South America, Central, East, Southern, and West Africa, and North America. IeDEA-SA currently includes a combined database of individual-level data on more than 640,000 adults and children on ART in 17 large collaborating sites in Lesotho, Malawi, Mozambique, South Africa, Zambia and Zimbabwe.

## TRACING-INFORMED STUDY IN FIVE SOUTHERN AFRICAN COUNTRIES

### Study population

Retrospective cohort study among CAYHIV aged ≤24 years initiating ART between 2004 and 2017 in seven ART cohorts in Lesotho, Malawi, Mozambique, Zambia and Zimbabwe.

### Tracing sample

A multi-country tracing study was conducted among all people living with HIV (PLWH) reported as LTFU from selected IeDEA-SA programs between October 2017 and November 2019 [16, 17]. From the traced sample of 3256 PLWH, we analysed a subsample of 680 CAYHIV with no recorded visit for ≥180 days or LTFU according to the sites’ definition and not transferred out or deceased. Tracing used text messages, phone calls and home visits, reported elsewhere [16, 17].

## LINKAGE-INFORMED STUDY IN WC, SOUTH AFRICA

### Study population

Retrospective cohort study among all CAYHIV who initiated ART aged ≤14 years between 2004 and 2019 at four IeDEA-SA WC sites [13, 14]. All sites are in Cape Town: two primary healthcare facilities (Khayelitsha and Gugulethu) and two tertiary hospitals (Tygerberg and Red Cross).

### Linking records to the Provincial Health Data Centre

We identified CAYHIV aged 0–14 years at ART start reported as LTFU (no recorded visit at clinic of ART initiation for ≥180 days before database closure and not recorded as transferred out or deceased, with the last visit date before meeting the LTFU definition deemed the LTFU date). The province uses unique patient identifiers in all public health services. Utilising the unique identifier, the Western Cape Provincial Health Data Centre (WCPHDC) [18] provided sites with additional data on lost patients, including any HIV or non-HIV-related encounters within the WC (health visits, pharmacy, laboratory tests, hospitalisation or death in a health facility). The de-identified linked data were shared with IeDEA-SA, allowing us to determine whether a patient had been hospitalised, died or was receiving routine HIV care at another facility within the WC and retained, or was truly lost.

## OUTCOMES

We defined

- **All-cause mortality:** as having a recorded death date at the clinic or confirmed to have died through tracing, with information obtained through interviewing close informants, relatives or caregivers or through linkage to the WCPHDC.
- **Self-transfer:** as either a patient/informant reporting (tracing) or having electronic evidence (linkage) of a visit at another facility, ART pick-up, laboratory test, or hospitalisation after being reported as LTFU at the original ART initiation facility.
- **True LTFU:** as previously reported LTFU with no evidence (reported or from linkage) of a healthcare visit or death.

## ANALYSIS

Analyses were performed in R version 4.2.2 [19] and Stata v17 (Stata Corp., College Station, TX, USA). We summarised patient characteristics and outcomes using proportions, medians, and interquartile ranges (IQRs) [20]. ‘Immune-suppression’ was based on the World Health Organisation definition, using CD4 cell count/μL (<350 cells/μL) for individuals aged ≥60 months and whichever of CD4 percentage (CD4% < 30 for those under 12 months, <25 for those aged 12–35 months and <20 for those aged 36–59 months) or count was available for those aged <60 months [21]. Follow-up time was defined as the time from the ART start date to:

- date of death for CAYHIV with a recorded death at the facility or found to have died through tracing or linkage,
- database closure date or tracing date for CAYHIV who were reported as in care at the facility or found to still be in care at the same facility on tracing,
- tracing or linkage date if found to have self-transferred and
- last contact date for CAYHIV who remained LTFU.

## CORRECTING MORTALITY ESTIMATES FOR LTFU

We compared Kaplan–Meier (K-M) outputs for six methods (3 ‘uncorrected’ and 3 ‘corrected’) with estimate mortality. ‘Corrected’ methods accounted for unascertained mortality in those reported as LTFU.

## ‘UNCORRECTED’ METHODS

These methods were:

1. Complete cases (CC): discarding observations for CAYHIV reported as LTFU.
2. Non-informative censoring (NIC): censoring CAYHIV reported as LTFU from last contact date. NIC is used in most analyses of program outcomes that do not trace LTFU patients. In this method, we imputed missing covariate data on immune-suppression (model explained in Supporting Information).
3. Multiple imputation (MI(naïve)) of survival times and event status using a joint modelling approach [22] with all measured variables included in the imputation model (Supporting Information). This method assumes mortality can be imputed without additional tracing/linkage data, but this may be problematic if outcomes differ between those lost and in care.

## ‘CORRECTED’ METHODS

These methods were:

- MI(asc.): multiple imputation after including the ascertained (asc.) data (outcomes from tracing/linkage) with indicators of missingness and ascertainment (successfully traced/linked) included in the imputation model [9].
- Conventional inverse probability weights (IPW), weighting those LTFU and traced/linked by the inverse probability of being found/linked to represent all those who are lost using a null logistic model (IPW(asc.,cw)) (Supporting Information).
- IPW(asc.,lw) as in (5) but the probability to be found modelled as: Two logistic regression models for demographic and clinic characteristics associated with being sampled for tracing if LTFU and characteristics associated with being found by the tracer (‘two-stage IPW’) for tracing. One logistic model weighted for demographic and clinic characteristics associated with being successfully linked as all LTFU patients were in the linked sample (‘one-stage IPW’) (Supporting Information).

We used weighted Cox regression using R package coxphw [23] to assess factors associated with all-cause mortality using uncorrected and corrected methods.

### ETHICS STATEMENT

IeDEA-SA cohorts have ethical approval to collect and transfer anonymised data through their respective Institutional Review Boards. IeDEA-SA data centre has approval from the University of Cape Town’s Human Research Ethics Committee to receive and analyse these de-identified data. Cohort data were linked to WCPHDC by a staff member who has permission to access identified patient data. The linked data were de-identified before transfer to IeDEA-SA data centre for analysis.

## RESULTS

### Patient characteristics in the five Southern African countries excluding South Africa (routine patients and tracing sample)

#### All routine patients (both retained and lost)

Among the 79,876 CAYHIV (33,187(42%) retained, 46,689(58%) reported as LTFU), 29% were males, with a median age at ART start of 18.7 (IQR: 7.9, 22.5) years (Table 1). Less than half were immune-suppressed at ART initiation, and 55% initiated ART between 2004 and 2013. At last visit, median age was 20.3 (IQR: 11.4, 23.9) years, with nearly 50% immune-suppressed and 44,588(56%) on ART for ≥12 months. Before tracing a sample of lost participants, there were 4164/33,187 (12.6%) reported deaths among patients retained in care. Compared with retained patients, those reported as LTFU were older at ART start (19.1 [IQR: 8.3, 22.5] vs. 17.9 [IQR: 7.4, 22.4] years), more likely immune-suppressed at ART start and last visit (53% vs. 30% and 57% vs. 38%, respectively), and less likely to have been on ART for ≥12 months at their last visit (54% vs. 58%, respectively). In a sensitivity analysis, we restricted to CAYHIV aged 0–14 years at last visit totalling 25,384. In this subset, among those who remained in care, mortality was 16.1% (1824/11,350) and 21.0% (43/205) among those who were lost and traceable.

#### Vital status confirmed (alive/dead) versus not confirmed (true LTFU) in the tracing sample

Among 680 patients reported as LTFU, 462/680 (68%; 39% males) were found (successfully traced) with vital status ascertained, and 218/680 (32%; 42% males) remained LTFU (Table 1). Of patients with ascertained vital status, 62/462 (13.4%) had died and the remainder were confirmed alive although their retention status could not always be ascertained.

The median (IQR) age at ART start was similar among CAYHIV whose vital status was and was not ascertained in the tracing sample. However, at last visit, patients with ascertained vital status were younger (17.6 [IQR: 6.3, 21.8] vs. 21.1 [IQR: 12.7, 24.6] years), more likely immune-suppressed (29% vs. 19%) and more likely to have been on ART for ≥12 months (56% vs. 29%) than those who could not be traced. Mortality was slightly higher among those reported as LTFU and traceable than those remaining in care (13.4% vs. 12.6%), with great variability across countries (Table 2).

#### Comparison of K-M curves and Cox-model estimates using uncorrected and corrected methods for incorporating ascertained mortality

**FIGURE 1:**
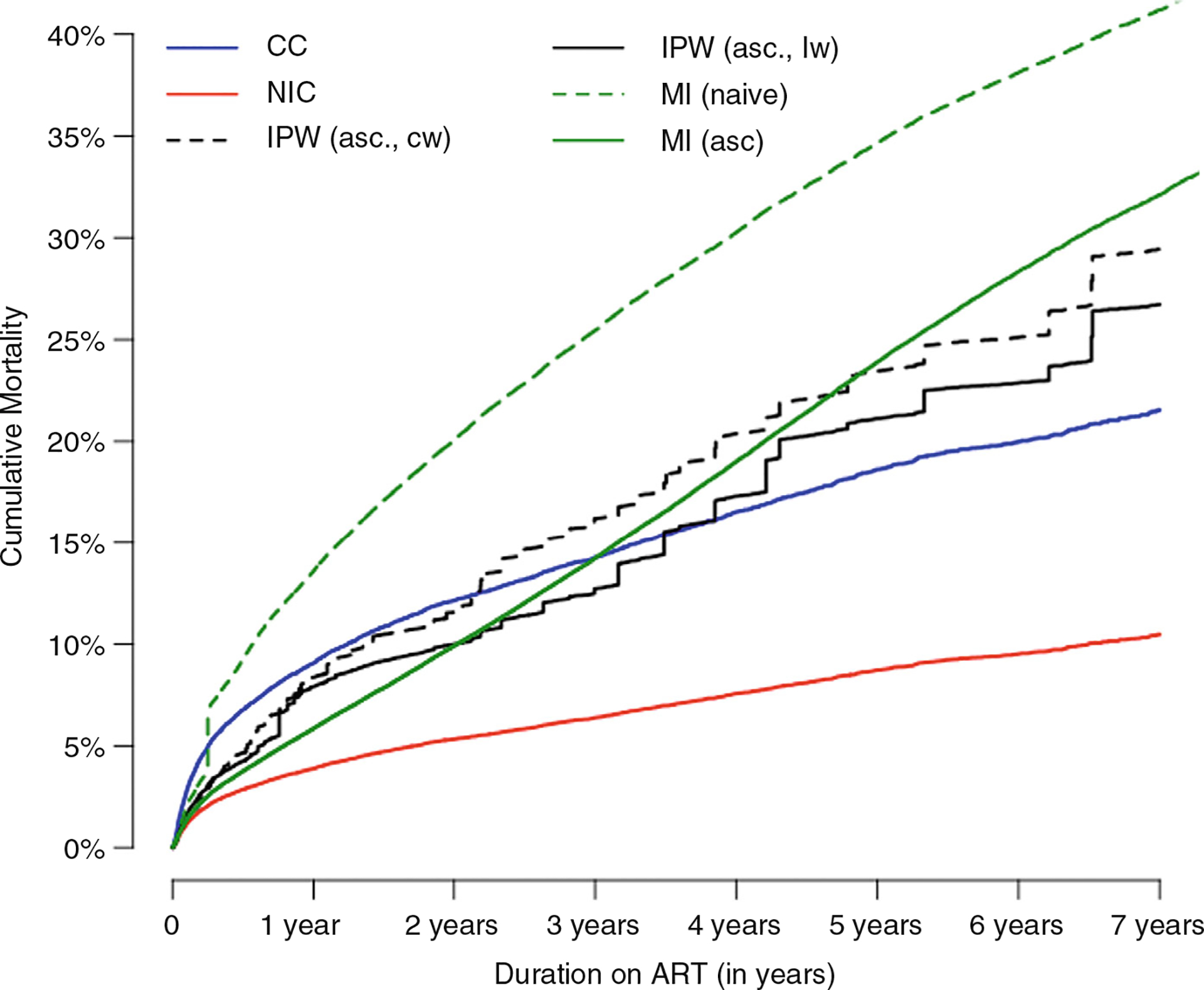
Comparison of Kaplan–Meier survival curves using naïve methods: Complete case (CC), non-informative censoring (NIC), inverse probability weighting (constant weights, IPW(asc.,cw)), inverse probability weighting (regression weights, IPW(asc.,lw)), multiple imputation, MI(naïve)) and corrected methods: Multiple imputation with ascertainment MI(asc.)) in Southern Africa data. Additional outcome ascertainment of those lost to follow-up was obtained through tracing. ART, antiretroviral therapy.

Corrected methods produced higher mortality rates than uncorrected methods except for the MI(naïve) method, which imputed high mortality in those LTFU and estimated the highest mortality (Figure 1). The NIC method yielded the lowest mortality estimates. Among the corrected methods, IPW(asc.,cw) yielded higher estimates than IPW (asc.,lw). MI(asc.) yielded similar estimates to IPW(asc.,lw) until 2 years, whereafter MI(asc.) estimates were consistently higher.

Estimates of associations with mortality from corrected methods were attenuated compared with uncorrected methods (Table 3) due to relative differences in mortality in traced versus retained patients for different patient characteristics. For example, the association between age <2 years at last visit with mortality versus age 2–<20 years was attenuated, as the relative mortality difference in the traced versus retained CAYHIV was greater for those aged 2–<20 years (Table S1). Similarly, the apparent lower mortality of recent ART initiation calendar years (2014–2017) versus earlier years was attenuated due to relatively more unreported deaths in recent years versus earlier years among those who were traced than those retained.

### Patient characteristics in Western Cape (routine patients and linked data)

#### All routine patients (both retained and lost)

Among 6728 CAYHIV (5008 [74%] retained, 1720 [26%] reported as LTFU) with age at ART start <15 years, 48% were males, with median age at ART start of 1.5 (IQR: 0.4, 5.5) years (Table 4). At ART initiation, over 50% were immune-suppressed. At last visit, median age was 7.2 (IQR: 2.4, 13.2) years, with a quarter immune-suppressed. Most (58%) CAYHIV initiated ART between 2004 and 2009 with nearly two-thirds on ART for ≥12 months. Before linkage, there were 468/5008 (9.4%) reported deaths among those retained in care. Compared with retained patients, fewer of those reported as LTFU were immune-suppressed at ART start (42% vs. 61%) and at last visit they were older (median age 10.9 [IQR: 4.6, 16.7] vs. 6.2 [IQR: 1.9, 12.0] years) with a greater proportion on ART for ≥12 months; 76% versus 54%.

#### Vital status confirmed (alive/dead) versus not confirmed (true LTFU) in linkage data

Of 1720 patients reported as LTFU, 1310 (76%) were successfully linked and 410 (24%) were truly lost with no further records in the WCPHDC (Table 4). Among successfully linked patients, 45/1310 (3.4%) had died, and 1265/1310 (97%) were receiving care within the WC, having self-transferred (802/1265 [63%]) or been hospitalised and were alive at discharge (463/1265 [37%]).

Compared with patients without linkable records, linkable patients were older (median age 2.1 [IQR: 0.6, 6.6] vs. 1.5 [IQR: 0.4, 5.0] years) and less likely immune-suppressed (37% vs. 57%) at ART start and last visit (median age 12.9 [IQR: 6.1, 18.2] vs. 5.5 [IQR: 2.5, 11.2] years; 24% vs. 33% immune-suppressed). More linkable than unlinkable patients were on ART for ≥12 months at last visit (82% vs. 59%). Mortality was higher among patients retained than those with vital status ascertained through linkage (9.4% vs. 3.4%) (Table 5).

#### Comparison of K-M curves and Cox-model estimates using uncorrected and corrected methods for incorporating ascertained mortality

**FIGURE 2:**
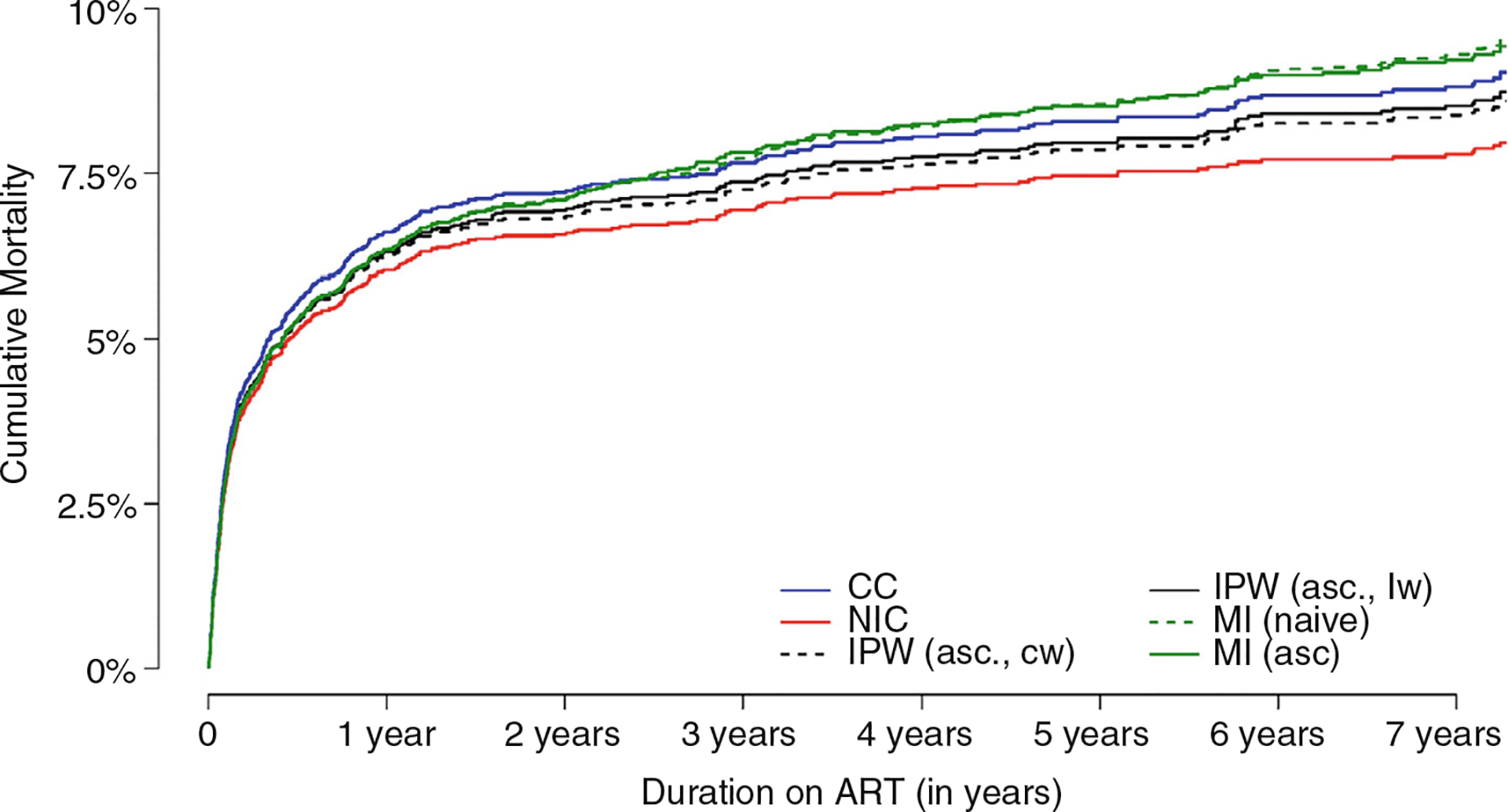
Comparison of Kaplan–Meier survival curves using naïve methods: Complete case (CC), non-informative censoring (NIC), inverse probability weighting (constant weights, IPW(asc.,cw)), inverse probability weighting (regression weights, IPW(asc.,lw)), multiple imputation, MI(naïve)) and corrected methods: Multiple imputation with ascertainment MI(asc.)) in Western Cape data. Additional outcome ascertainment of those lost to follow-up was obtained through linkage to the WCPHDC. ART, antiretroviral therapy.

The NIC method consistently yielded the lowest estimates, despite low mortality in those reported as LTFU and linked (Figure 2). Due to high early mortality in facilities and few deaths ascertained through linkage, the CC method yielded higher estimates than corrected methods in the first 2 years. The MI (naïve) method yielded the highest mortality estimates at longer ART durations among all uncorrected methods.

Overall, associations with mortality from corrected methods were similar to uncorrected methods (Table 6), as few extra deaths were identified from linkage. Nonetheless, the association of immune-suppression with mortality was attenuated in corrected versus uncorrected methods because mortality was relatively higher among patients who are immune-suppressed and retained versus linked patients than those non-immune-suppressed (Table S1).

## DISCUSSION

We believe this is the first multi-country study comprehensively examining outcomes among CAYHIV reported as LTFU using tracing and linkage. Correcting for true outcomes (death or self-transfer) impacted mortality and retention estimates, with substantial variation between the tracing and linkage approaches. Ascertained mortality was higher among lost and traceable CAYHIV and lower among lost and linkable CAYHIV than those retained in care. Younger age, immune-suppression at last visit, and recent calendar years of ART initiation were associated with higher mortality in tracing and linkage approaches, although these associations were attenuated using corrected methods in the tracing approach.

Our study confirms previous findings that reported LTFU is non-ignorable, as patients reported as lost were identified as deceased or self-transferred, and demonstrates the complementarity of tracing and linkage in outcome ascertainment [9, 17, 24–26]. In addition, the reasons for reported LTFU among CAYHIV may be different from those of adults. CAYHIV reported as LTFU and traced were more likely to have died and less likely to have self-transferred than those who were linked (mortality: tracing vs. linkage: 13% vs. 3%; self-transfer: tracing vs. linkage: 21% vs. 47%). The resource-intensive nature of tracing, using text messages, phone calls and home visits, probably allowed for a more thorough investigation and identification of deaths than linkage. However, although tracers can ascertain community deaths that are not reported to health facilities, they may not get accurate confirmation about a child’s health visits compared with using electronic health records. In contrast, the WCPHDC links unique patient identifiers to health service data [18] and is more likely to find live patients accessing care within the health system, but unlikely to identify out-of-facility deaths, explaining the low mortality. Nevertheless, one site, a referral hospital where very sick and/or young children initiate ART, with high mortality, had more deaths among lost and linkable CAYHIV than those retained. Unlike with adults, we could not link CAYHIV data to the National Population Register to ascertain missing out-of-facility deaths as few children have national identity numbers recorded in the WCPHDC [24]. Also, linkage was only possible for health visits in the WC, and we missed CAYHIV who migrated outside the WC [24]. Although linkage likely under-ascertained deaths in those LTFU, there may also be lower mortality in the WC due to better healthcare access and nutrition with less poverty than other countries.

Although mortality was higher among CAYHIV reported as LTFU and traced compared with those retained [16, 17, 25–28], previous studies have reported even higher mortality estimates in those LTFU [1, 3, 29]. In rural Tanzania, 40% of adults living with HIV who were reported LTFU and traced, had died [27]. Nonetheless, there was considerable observed variation between countries in our study suggesting that it may not be appropriate to apply mortality corrections for non-ignorable reported LTFU from one region, population or period to another, highlighting the need for updated context-specific estimates. Our sensitivity analysis restricting to only children aged ≤14 years (Supporting Information) revealed similar results as the main study including all CAYHIV (0–24 years).

The results from comparison of six statistical methods also varied between tracing and linkage approaches because some methods are inappropriate as their strict assumptions may not be met. Using tracing, corrected mortality estimates were higher than uncorrected, particularly after 2 years on ART, whereas the reverse was true using linkage due to low mortality in linked patients. The uncorrected NIC method yielded the lowest estimates for tracing and linkage, implying that without additional outcome ascertainment among those reported as LTFU (irrespective of the proportion ascertained), program-level mortality estimates will be underestimated, as has been found by other studies [2, 9, 27], whereas the CC method yielded higher mortality in the first 2 years of ART for tracing and linkage, this method is biased, especially with non-ignorable LTFU, due to excluding observations from patients reported as LTFU [30, 31]. Similarly, the MI(naïve) method, which yielded the highest mortality in both approaches, is biased because it does not incorporate information about missingness or the tracing and linkage processes [9]. The high estimated mortality may be driven by the imputation of deaths in those LTFU who are young and severely ill, as those retained with similar characteristics have the highest mortality. If the missingness process does not depend on the outcome, and we can model mortality among those lost using data from those retained in care, then MI(naïve) may provide correct results.

IPW methods yielded varying results. Logistic weights (IPW(asc.,lw)) produced lower mortality estimates in the tracing, whereas constant weights (IPW(asc.,cw)) did in the linkage approach. IPW(asc.,cw) has been widely used in other studies with consistent estimates [2, 3, 32]. However, if patient characteristics of those included in the tracing sample differ from those not included, or patients found by the tracer differ from those not found, as was the case in our study (Table 1), results may be biased [33]. IPW(asc.,lw) is then more appropriate but relies on correctly-specified models to derive weights [9, 27]. Overall, estimates between the uncorrected and corrected methods were only slightly improved in the linkage approach because we only ascertained additional deaths if they occurred in health facilities in the province, where a small proportion of all child deaths occur [34]. Given the low number of additionally ascertained deaths in the linkage data, IPW(asc.,lw) may yield inconsistent estimates because only a small number of outcome-covariate combinations was used to construct the weights (i.e., weights are based only on the few covariate values available among those identified as deceased). Schomaker et al. showed that MI(asc.) was the most stable and appropriate method for low levels of ascertainment in their analysis of adult data [9] and is thus likely the most robust method for updating mortality estimates using the linkage approach in our study, given the low level of mortality ascertainment, whereas the most robust method using tracing data would be MI(asc.) or IPW(asc.,lw). Mortality estimates solely derived from facility records without additional outcome ascertainment among individuals reported as LTFU are likely to be underestimated. This is because LTFU may obscure high rates of unreported mortality as seen in our studies. Hence, it is imperative for programs to endeavour to incorporate tracing or linkage efforts where feasible to estimate mortality in this group and pool these estimates with those at the facility for accurate reporting. Additionally, using suitable analytical methods to aggregate these estimates is essential for precise program-level estimation and reporting.

Risk factors for mortality were consistent in tracing and linkage approaches but with some differences between uncorrected and corrected methods due to relative differences in the proportion deceased among the retained and traced/linked CAYHIV with different characteristics. The finding of relatively fewer unreported deaths in traced younger CAYHIV versus older CAYHIV compared with those remaining in care may partly be because health services may ascertain and confirm deaths through caregivers as part of routinely collected data among younger children, which is less feasible for older children and young adults who are less reliant on caregivers [35, 36]. Attenuation of the effect of recent ART initiation calendar years in corrected methods suggests that while mortality in recent years may have declined, part of the decline may be due to higher rates of reported LTFU masking under-ascertained mortality.

This is the first large multi-country study comparing tracing and linkage approaches with ascertain outcomes among CAYHIV reported as LTFU and using these data to compare statistical methods for estimating program-level mortality among CAYHIV. However, there are several limitations. The tracing exercise included adults and children so the number of CAYHIV reported as LTFU in the tracing sample was limited. Nonetheless, these CAYHIV were selected to represent all lost patients in the relevant age groups of the respective cohorts [17]. The outcomes could only be ascertained among 68% of those traced and their outcomes may differ from those not successfully traced. Linkage was limited to health service encounters within the WC, potentially resulting in an underestimation of outcomes, although the corrected methods accounted for this potential under-ascertainment by weighting patients successfully linked to represent all who were not linked.

## CONCLUSIONS

This study highlights the importance and complementarity of tracing and linkage approaches to ascertain outcomes among CAYHIV reported as LTFU and confirms that LTFU is non-ignorable. Among those reported as LTFU, tracing identified substantial mortality while linkage showed that a large proportion were self-transfers but was unable to identify out-of-facility deaths. Incorporating additional data on mortality in those reported as LTFU is context-specific and it may not be appropriate to generalise correction of outcome estimates from one region or cohort to another.

## Supplementary Material

Supplementary information

Supplementary tables

Supplementary figures

## Figures and Tables

**TABLE 1:** Patient characteristics at antiretroviral therapy start and last visit in routine and tracing data.

Variable	All routine patients (*N* = 79,876)	Retained in care (*N* = 33,187)	Lost patients (*N* = 46,689)	Tracing sample^a^ (from lost) (*N* = 680)	Vital status confirmed (alive/dead) (*N* = 462)	Vital status not confirmed (remained LTFU) (*N* = 218)
	*n* (%)	*n* (%)	*n* (%)	*n* (%)	*n* (%)	*n* (%)
Sex						
Male	23,316 (29.2)	10,246 (30.9)	13,070 (28.0)	273 (40.2)	182 (39.4)	91 (41.7)
Age at ART start, median (IQR) years	18.67 (7.90, 22.46)	17.90 (7.41, 22.37)	19.11 (8.26, 22.52)	16.57 (4.73, 21.10)	14.38 (4.31, 20.68)	14.13 (3.82,21.08)
Age at ART start, years						
0–<2	7734 (9.7)	3452 (10.4)	4282 (9.2)	114 (16.7)	75 (16.2)	39 (17.9)
2–<10	16,286 (20.4)	7066 (21.3)	9220 (19.8)	136 (20.0)	92 (19.9)	44 (20.2)
10–<20	21,099 (26.4)	8960 (27.0)	12,139 (26.0)	209 (30.7)	144 (31.2)	65 (29.8)
20–24	34,745 (43.5)	13,709 (41.3)	21,036 (45.1)	209 (30.7)	142 (30.7)	67 (30.7)
Age at last visit, median (IQR) years	20.33 (11.43, 23.95)	20.09 (11.22, 24.00)	21.03 (12.67, 24.59)	17.00 (5.40, 21.66)	17.58 (6.31, 21.80)	21.08 (12.74, 24.62)
Age at last visit, years						
0–<2	3088 (3.9)	1360 (4.1)	1728 (3.7)	72 (10.6)	48 (10.4)	24 (11.0)
2–<10	14,313 (17.9)	5984 (18.0)	8329 (17.8)	154 (22.7)	100 (21.7)	54 (24.8)
10–<20	21,370 (26.8)	9158 (27.6)	12,212 (26.2)	203 (29.9)	141 (30.5)	62 (28.4)
≥20	41,105 (51.5)	16,685 (50.3)	24,420 (52.3)	251 (36.9)	173 (37.5)	78 (35.8)
Immune-suppression at last visit						
No	1516 (1.9)	359 (1.1)	1157 (2.6)	82 (12.1)	61 (13.2)	21 (9.6)
Yes	39,069 (48.9)	12,597 (38.0)	26,472 (56.7)	177 (26.0)	135 (29.2)	42 (19.3)
Missing	39,291 (49.2)	20,231 (61.0)	19,060 (40.8)	421 (61.9)	266 (57.6)	155 (71.1)
Year of ART start						
2004–2013	44,268 (55.4)	16,452 (49.6)	27,816 (59.6)	127 (18.7)	96 (20.7)	31 (14.2)
2014–2015	18,318 (22.9)	6170 (18.6)	12,148 (26.0)	372 (54.7)	241 (52.2)	131 (60.1)
2016–2017	17,290 (21.7)	10,565 (31.8)	6725 (14.4)	181 (26.6)	125 (27.1)	56 (25.7)
Duration on ART at last visit, months						
0–<6	27,589 (34.5)	10,906 (32.9)	16,683 (35.7)	263 (38.7)	141 (30.5)	114 (52.3)
6–<12	7699 (9.6)	3011 (9.1)	4688 (10.0)	100 (14.7)	63 (13.6)	41 (18.8)
≥12	44,588 (55.9)	19,270 (58.1)	25,318 (54.2)	317 (46.6)	258 (55.8)	63 (28.9)
Country						
Country A	2251 (2.8)	868 (2.6)	1383 (3.0)	38 (5.2)	25 (5.4)	13 (6.0)
Country B	19,755 (24.7)	10,353 (31.2)	9402 (20.1)	265 (36.1)	181 (39.2)	84 (38.5)
Country C	5012 (6.3)	742 (2.2)	4270 (9.2)	131 (17.9)	109 (23.6)	22 (10.1)
Country D	40,550 (50.8)	14,010 (42.2)	26,540 (56.8)	136 (18.5)	49 (10.6)	87 (5.5)
Country E	12,308 (15.4)	7214 (21.7)	5094 (10.9)	110 (15.0)	98 (21.2)	12 (5.5)

Abbreviations: ART, antiretroviral therapy; CAYHIV, Children, adolescents and young adults; LTFU, loss to follow-up.

a Tracing sample forms part of a larger traced sample (*n* = 3256) that was sampled and traced including adult patients [17].

**TABLE 2:** Summary of the data used in the tracing analysis.

Country	CAYHIV	LTFU (*n* (%))	Traced^a^ (*n* (%))	Non-LTFU died (%)	Traceable LTFU died (%)
Country A	2251	1383 (61.4)	38 (1.7)	19.6	4.8
Country B	19,755	9402 (47.6)	265 (1.3)	7.7	41.9
Country C	5012	4270 (85.2)	131 (2.6)	63.8	30.6
Country D	40,550	26,540 (65.5)	136 (0.3)	14.3	8.1
Country E	12,308	5094 (41.4)	110 (0.9)	10.1	14.5
Total	79,876	46,689 (58.5)	680 (0.9)	12.6	13.4

Abbreviations: CAYHIV, children, adolescents and young adults; LTFU, loss to follow-up.

a Tracing sample forms part of a larger traced sample (*n* = 3256) that was sampled and traced including adult patients [17].

**TABLE 3:** Adjusted Cox regression models of mortality after correcting for LTFU among those who were traced and found using different weighting methods.

Patient characteristics	CC	NIC	MI(naïve)	IPW(asc.,cw)	IPW(asc.,lw)	MI(asc)
	aHR^a^ (95% CI)	aHR^a^ (95% CI)	aHR^a^ (95% CI)	aHR^a^ (95% CI)	aHR^a^ (95% CI)	aHR^a^ (95% CI)
Sex						
Male	Ref	Ref	Ref	Ref	Ref	Ref
Female	0.93 (0.85, 1.01)	0.87 (0.80, 0.95)	1.04 (0.98, 1.10)	1.02 (0.94, 1.09)	1.02 (0.94, 1.09)	1.02 (0.94, 1.09)
Age at last visit, years						
0–<2	Ref	Ref	Ref	Ref	Ref	Ref
2–<10	0.09 (0.08, 0.11)	0.05 (0.04, 0.06)	0.22 (0.20, 0.24)	0.20 (0.18, 0.22)	0.20 (0.18, 0.22)	0.20 (0.18, 0.22)
10–<20	0.05 (0.04, 0.06)	0.03 (0.03, 0.04)	0.14 (0.13, 0.16)	0.13 (0.12, 0.15)	0.13 (0.12, 0.15)	0.13 (0.12, 0.15)
≥20	0.08 (0.07, 0.10)	0.05 (0.04, 0.06)	0.19 (0.17, 0.21)	0.18 (0.16, 0.20)	0.18 (0.16, 0.20)	0.18 (0.16, 0.20)
Immune-suppression at last visit						
No	Ref	Ref	Ref	Ref	Ref	Ref
Yes	1.14 (0.92, 1.42)	1.98 (1.59, 2.45)	1.02 (0.91, 1.15)	1.07 (0.96, 1.18)	1.07 (0.96, 1.18)	1.07 (0.96, 1.18)
Year of ART start						
2004–2013	Ref	Ref	Ref	Ref	Ref	Ref
2014–2015	0.30 (0.26, 0.35)	0.33 (0.28, 0.38)	0.91 (0.84, 0.97)	0.83 (0.75, 0.92)	0.83 (0.75, 0.92)	0.83 (0.75, 0.92)
2016–2019	0.14 (0.11, 0.18)	0.20 (0.15, 0.26)	0.98 (0.89, 1.07)	0.79 (0.73, 0.85)	0.79 (0.73, 0.85)	0.79 (0.73, 0.85)

Abbreviations: CC, complete case; CI, confidence interval; IPW (asc.,cw), Inverse Probability Weighting with constant weights; IPW (asc.,lw), Inverse Probability Weighting with logistic weights; MI (naïve), multiple imputations before ascertainment; MI(asc), multiple imputations with ascertainment; NIC, non-informative censoring.

a Adjusted for country where health facility is located.

**TABLE 4:** Patient characteristics at antiretroviral therapy start and last visit among children and adolescents who initiated treatment between 2004 and 2019 in Western Cape, South Africa.

Variable	All routine patients (*N* = 6728)	Retained in care (*N* = 5008)	Lost patients (*N* = 1720)	Vital status confirmed (alive/dead) (*N* = 1310)	Vital status not confirmed (remained LTFU) (*N* = 410)
	*n* (%)	*n* (%)	*n* (%)	*n* (%)	*n* (%)
Sex					
Male	3258 (48.4)	2443 (48.8)	815 (47.4)	626 (47.8)	189 (46.1)
Age at ART start, median (IQR) years	1.54 (0.41, 5.47)	1.42 (0.38, 5.26)	1.88 (0.54, 6.22)	2.07 (0.62, 6.61)	1.52 (0.41, 5.02)
Age at ART start, years					
0–<2	3723 (55.3)	2841 (56.7)	882 (51.3)	646 (49.3)	236 (57.6)
2–<10	2381 (35.4)	1746 (34.9)	635 (36.9)	498 (38.0)	137 (33.4)
10–<15	624 (9.3)	421 (8.4)	203 (11.8)	166 (12.7)	37 (9.0)
Immune-suppression at ART start					
No	2165 (32.3)	1392 (27.8)	773 (44.9)	670 (51.2)	103 (25.1)
Yes	3774 (56.1)	3052 (60.9)	722 (42.0)	488 (37.3)	234 (57.1)
Missing	789 (11.7)	564 (11.3)	225 (13.1)	152 (11.6)	73 (17.8)
Age at last visit, median (IQR) years	7.20 (2.39, 13.20)	6.18 (1.87, 12.0)	10.85 (4.61, 16.65)	12.92 (6.05, 18.21)	5.48 (2.48, 11.21)
Age at last visit, years					
0–<2	1514 (22.5)	1314 (26.2)	200 (11.6)	111 (8.5)	89 (21.7)
2–<10	2642 (39.3)	2036 (40.7)	606 (35.2)	399 (30.5)	207 (50.5)
10–<15	1589 (23.6)	1163 (23.2)	426 (24.8)	341 (26.0)	85 (20.7)
≥15	983 (14.6)	495 (9.9)	488 (28.4)	459 (35.0)	29 (7.1)
Immune-suppression at last visit					
No	3675 (54.6)	2580 (51.5)	1095 (63.7)	868 (66.3)	227 (55.4)
Yes	1759 (26.1)	1305 (26.1)	454 (26.4)	318 (24.3)	136 (33.2)
Missing	1294 (19.2)	1123 (22.4)	171 (9.9)	124 (9.5)	47 (11.5)
Viral load at last visit					
<400	2874 (42.7)	2240 (44.7)	634 (36.9)	486 (37.1)	148 (36.1)
≥400	2661 (39.6)	2027 (40.5)	634 (36.9)	482 (36.8)	152 (37.1)
Missing	1193 (17.7)	741 (14.8)	452 (26.3)	342 (26.1)	110 (26.8)
Year of ART start					
2004–2008	2026 (30.1)	1604 (32.0)	422 (24.5)	353 (27.0)	69 (16.8)
2009–2013	1852 (27.5)	1410 (28.2)	442 (25.7)	393 (30.0)	49 (12.0)
2014–2019	1400 (20.8)	998 (19.9)	402 (23.4)	295 (22.5)	107 (26.1)
2013–2015	838 (12.5)	553 (11.0)	285 (16.6)	181 (13.8)	104 (25.4)
2016–2019	612 (9.1)	443 (8.9)	169 (9.8)	88 (6.7)	81 (19.8)
Duration on ART at last visit, months					
0–<6	1156 (17.2)	988 (19.7)	168 (9.8)	90 (6.9)	78 (19.0)
6–<12	1565 (23.3)	1328 (26.5)	237 (13.8)	145 (11.1)	92 (22.4)
≥12	4007 (59.6)	2692 (53.8)	1315 (76.1)	1075 (82.1)	240 (58.5)
Site					
Site 1	747 (11.1)	344 (6.9)	403 (23.4)	216 (16.5)	187 (45.6)
Site 2	1961 (29.2)	1296 (25.9)	665 (38.7)	645 (49.2)	20 (4.9)
Site 3	2378 (35.3)	2033 (40.6)	345 (20.1)	146 (11.2)	199 (48.5)
Site 4	1642 (24.4)	1335 (26.7)	307 (17.9)	303 (23.1)	4 (1.0)

Abbreviations: ART, antiretroviral therapy; CAYHIV, children, adolescents and young adults; LTFU, loss to follow-up.

**TABLE 5:** Summary of the data used in the linkage analysis.

Cohort	CAYHIV	LTFU/ linked (n (%))	Non-LTFU died (%)	Linkable LTFU died (%)
Site 1	747	403 (54.0)	5.8	1.2
Site 2	1961	665 (33.9)	2.0	3.9
Site 3	2378	345 (14.5)	14.2	1.2
Site 4	1642	307 (18.7)	10.0	3.3
Total	6728	1720 (25.6)	9.4	3.4

**TABLE 6:** Adjusted Cox regression models of mortality after correcting for LTFU among those who were linked and found using different weighting methods.

Patient characteristics	CC	NIC	MI (naive)	IPW (asc., cw)	IPW (asc., lw)	MI (asc)
	aHR^a^ (95% CI)	aHR^a^ (95% CI)	aHR^a^ (95% CI)	aHR^a^ (95% CI)	aHR^a^ (95% CI)	aHR^a^ (95% CI)
Sex						
Male	Ref	Ref	Ref	Ref	Ref	Ref
Female	1.06 (0.88, 1.28)	1.08 (0.90, 1.31)	1.06 (0.85, 1.32)	1.10 (0.93, 1.31)	1.10 (0.93, 1.31)	1.10 (0.93, 1.31)
Age at last visit, years						
0–<2	Ref	Ref	Ref	Ref	Ref	Ref
2–<10	0.21 (0.16, 0.28)	0.20 (0.15, 0.26)	0.20 (0.16, 0.25)	0.17 (0.11, 0.19)	0.15 (0.11, 0.19)	0.15 (0.11, 0.19)
10–<15	0.10 (0.07, 0.17)	0.09 (0.06, 0.14)	0.06 (0.03, 0.10)	0.05 (0.03, 0.06)	0.04 (0.03, 0.06)	0.04 (0.03, 0.06)
≥15	0.05 (0.03, 1.00)	0.03 (0.02, 0.06)	0.03 (0.02, 0.05)	0.03 (0.01, 0.03)	0.02 (0.01, 0.03)	0.02 (0.01, 0.03)
Immune-suppression at last visit						
No	Ref	Ref	Ref	Ref	Ref	Ref
Yes	12.19 (9.30, 15.96)	11.40 (8.72, 14.90)	6.34 (4.92, 8.18)	7.89 (6.54, 10.32)	8.22 (6.54, 10.32)	8.22 (6.54, 10.32)
Year of ART start						
2004–2006	Ref	Ref	Ref	Ref	Ref	Ref
2007–2009	0.68 (0.55, 0.83)	0.65 (0.53, 0.80)	0.74 (0.58, 0.95)	0.66 (0.55, 0.82)	0.67 (0.55, 0.82)	0.67 (0.55, 0.82)
2010–2012	0.45 (0.33, 0.62)	0.40 (0.29, 54)	0.61 (0.47, 0.78)	0.44 (0.35, 0.64)	0.47 (0.35, 0.64)	0.47 (0.35, 0.64)
2013–2015	0.44 (0.29, 0.68)	0.38 (0.25, 0.58)	0.68 (0.47, 0.97)	0.40 (0.27, 0.61)	0.40 (0.27, 0.61)	0.40 (0.27, 0.61)
2016–2019	0.15 (0.07, 0.31)	0.15 (0.07, 0.31)	0.41 (0.25, 0.65)	0.21 (0.16, 0.46)	0.27 (0.16, 0.46)	0.27 (0.16, 0.46)

Abbreviations: CC, complete case; CI, confidence interval; IPW (asc.,cw), Inverse Probability Weighting with constant weights; IPW (asc.,lw), Inverse Probability Weighting with logistic weights; MI (naïve), multiple imputations before ascertainment; MI(asc), multiple imputations with ascertainment; NIC, non-informative censoring.

a Adjusted for site where patient initiated ART.
