# Supplementary material for: Correcting mortality estimates among children and youth on antiretroviral therapy in southern Africa: A comparative analysis between a multi-country tracing study and linkage to a health information exchange
Source: Trop Med Int Health. Author manuscript; Available in PMC 2025 Feb 5. (PMC11795028; doi:10.1111/tmi.14030)
Supplement: Supplementary information [file NIHMS2052004-supplement-Supplementary_information.docx]

**Supplementary information regarding the imputation models.**

In all our imputation models, we applied the Amelia II method for imputation, utilizing five imputation models and then combined the results using Rubin's rules.

Our imputation process:

1. **Uncorrected Non-Informative Censoring (NIC) Models**: We imputed missing data relating to immune-suppression at the last visit. The imputation model included complete variables of age at the last visit, sex, year of ART initiation, the country where the ART program was located, censored mortality and survival time outcomes, and the partially observed immune-suppression variable.
2. **Multiple imputation (MI (naïve)) model**: We imputed all missing immune-suppression data for all CAYHIV, mortality and time-to-event data for those reported as LTFU. The imputation model included complete variables of age at the last visit, sex, year of ART initiation, the country where the ART program was situated, the incomplete immune-suppression variable, and the partially observed mortality and survival time outcomes.
3. **Inverse probability weighting (IPW) and Multiple imputation with ascertainment (MI (asc))**: We conducted imputation for missing immune-suppression data among all CAYHIV, incorporating information obtained from tracing and linkage. The imputation model included complete variables of age at the last visit, sex, year of ART initiation, the country where the ART program was located, mortality and survival time outcomes, indicators for missing data, and indicators for ascertainment.

**Supplementary information regarding the inverse probability (IPW) models.**

1. **Inverse probability weighting model (IPW, constant weights):** This was a null logistic regression model with no covariates weighting those LTFU and traced/linked by the inverse probability of being found/linked to represent all those who are lost.
2. **Inverse probability weighting model (IPW, logistic weights):** This model included two logistic models. The first model whose outcome was the probability of being sampled for tracing was adjusted for patient characteristics of age at the last visit, sex, year of ART initiation, and the country where the ART program was located. The second logistic model whose outcome was the probability of being found by the tracer or being successfully linked was adjusted for patient characteristics of tracing: CD4 count at last visit, duration on ART, year of ART start, location of the clinic site (rural vs urban), level of care (hospital or health centre). **Note**: The linkage study only involved the logistic regression model of being successfully linked using patient characteristics since linkage was attempted for all the patients reported as LTFU.
