# Supplementary material for: Correcting mortality estimates among children and youth on antiretroviral therapy in southern Africa: A comparative analysis between a multi-country tracing study and linkage to a health information exchange
Source: Trop Med Int Health. Author manuscript; Available in PMC 2025 Feb 5. (PMC11795028; doi:10.1111/tmi.14030)
Supplement: Supplementary tables [file NIHMS2052004-supplement-Supplementary_tables.docx]

**Supplementary Table 1: Relative differences in mortality between traced/linked patients and retained patients by patient characteristics.**

| **Patient characteristics** | **Percent deceased** | |  | **Percent deceased** | |
| --- | --- | --- | --- | --- | --- |
|  | **Retained (%)** | **Traced (%)** |  | **Retained (%)** | **Linked (%)** |
| **Sex** |  |  |  |  |  |
| Male | 7.1 | 12.1 | Male | 8.9 | 2.7 |
| Female | 4.5 | 7.1 | Female | 9.8 | 2.5 |
| **Age at last visit, years** |  |  |  |  |  |
| 0-<2 | 20.4 | 23.6 | 0-<2 | 24.5 | 4.5 |
| 2-<10 | 6.1 | 13.0 | 2-<10 | 4.6 | 1.3 |
| 10-<20 | 4.4 | 6.9 | 10-≤15 | 3.1 | 1.9 |
| ≥20 | 4.3 | 4.4 | >15 | 3.2 | 4.1 |
| **Immune-suppression at last visit** |  |  |  |  |  |
| No | 7.8 | 4.9 | No | 3.1 | 1.4 |
| Yes | 6.4 | 9.0 | Yes | 28.0 | 5.7 |
| **Year of ART start** |  |  |  |  |  |
| 2004-2013 | 8.1 | 12.6 | 2004-2006 | 13.3 | 2.6 |
| 2014-2015 | 2.4 | 7.3 | 2007-2009 | 11.4 | 4.5 |
| 2016-2017 | 0.9 | 10.5 | 2010-2012 | 5.9 | 1.7 |
|  |  |  | 2013-2015 | 4.9 | 1.1 |
|  |  |  | 2016-2019 | 1.6 | 2.4 |
| **Duration on ART, months** |  |  |  |  |  |
| 0-5 | 8.0 | 10.6 | 0-5 | 33.0 | 4.2 |
| 6-24 | 5.3 | 19.1 | 6-24 | 6.1 | 2.5 |
| ≥24 | 3.6 | 13.6 | ≥24 | 2.3 | 2.4 |
