# Supplementary material for: Correcting mortality estimates among children and youth on antiretroviral therapy in southern Africa: A comparative analysis between a multi-country tracing study and linkage to a health information exchange
Source: Trop Med Int Health. Author manuscript; Available in PMC 2025 Feb 5. (PMC11795028; doi:10.1111/tmi.14030)
Supplement: Supplementary figures [file NIHMS2052004-supplement-Supplementary_figures.docx]

**Supplementary Figure 1:** Sensitivity analysis restricted to children aged 0-14 years in the tracing study.

: Comparison of Kaplan Meier survival curves using naïve methods: complete case (CC), non-informative censoring (NIC), inverse probability weighting (constant weights, IPW(asc.,cw)), inverse probability weighting (regression weights, IPW(asc.,lw)), multiple imputation, MI(naïve)) and corrected methods: multiple imputation with ascertainment MI(asc.)) in Southern Africa data. Additional outcome ascertainment of those lost to follow-up was obtained through tracing.


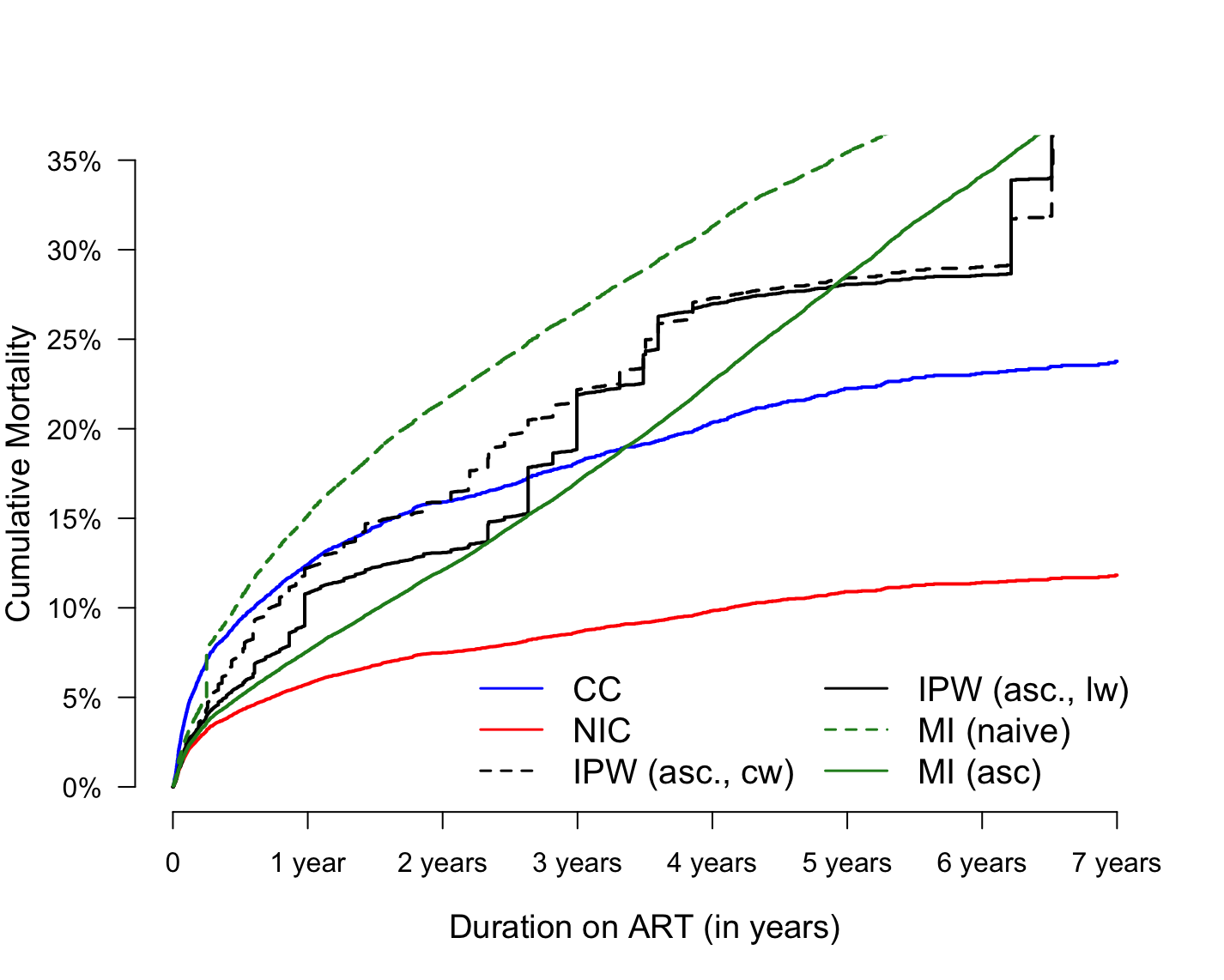


**References**

1. Ballif, M., et al., *Tracing people living with HIV who are lost to follow-up at ART programs in Southern Africa: A sampling-based cohort study in six countries.* Clinical infectious diseases, 2021.
